# Tuberculosis treatment outcomes and their determinants among patients attending Hargeisa TB Hospital: a five-year retrospective study

**DOI:** 10.11604/pamj.2025.52.27.48370

**Published:** 2025-09-18

**Authors:** Ahmed Ibrahim Farah, Ahmed Ahmed, Jama Mohamed, Abraham Nigussie Mekuria

**Affiliations:** 1 College of Health and Medical Sciences, University of Hargeisa, Hargeisa, Somaliland,; 2 National TB Program, Ministry of Health Development, Hargeisa, Somaliland,; 3 Doodi Hospital, Hargeisa, Somaliland,; 4 Hargeisa TB Hospital Data Management, Hargeisa, Somaliland,; 5 Faculty of Statistics and Data Science, University of Hargeisa, Hargeisa, Somaliland,; 6 Department of Pharmacology, School of Pharmacy, College of Health and Medical Sciences, Haramaya University, Harar, Ethiopia

**Keywords:** Tuberculosis, treatment outcome, age, HIV-coinfection, Hargeisa, Somaliland

## Abstract

**Introduction:**

tuberculosis (TB) is one of the major public health threats and the leading infectious disease worldwide. Assessing TB treatment outcomes and associated risk factors is crucial for effective treatment and control. This study aimed to investigate TB treatment outcomes and their related factors in Hargeisa, Somaliland.

**Methods:**

a five-year, hospital-based retrospective study was conducted at Hargeisa TB Hospital, Somaliland, from January 1^st^, 2019, to December 31^st^, 2023. A total of 6127 registered patients, including only 6069 TB patients with complete information, were included in the study. Demographic, clinical, and treatment characteristics of the study participants were gathered from the TB register using a pretested structured data abstraction template. Data were entered and analyzed using R 4.2.2 software, with a p-value < 0.05 considered statistically significant.

**Results:**

the overall successful treatment outcome was 81.2%. Among all TB patients, 31.9% of males and 21.8% of females completed their treatment. Younger patients (<20 years) showed a higher likelihood of success, as indicated by adjusted odds ratio (AOR= 2.89, 95% CI: 2.35-3.56), followed by patients aged 20-40 (AOR= 2.43, 95% CI: 1.98-2.98) and 40-60 (AOR= 1.54, 95% CI: 1.23-1.93). Patients with extrapulmonary tuberculosis (EPTB) had lower odds of successful treatment compared to those with pulmonary TB (AOR= 0.75, 95% CI: 0.66-0.86). Human Immunodeficiency virus (HIV)- negative patients were more likely to achieve a successful outcome (AOR= 2.75, 95% CI: 1.82-4.10), and the likelihood of success increased progressively over the years, with 2023 showing the highest odds (AOR= 2.66, 95% CI: 2.14-3.30).

**Conclusion:**

the overall treatment outcome was below the World Health Organization (WHO) target (90%). Therefore, the study recommends improvements in TB treatment strategies, focusing on at-risk groups for less treatment success, including older individuals and HIV-coinfected TB patients.

## Introduction

Globally, TB continues to pose a serious threat to public health, with developing nations bearing the majority of the burden. According to the WHO, TB was the leading cause of mortality from a single infectious agent, surpassing coronavirus disease of 2019 (COVID-19). In 2023, there were 8.2 million newly diagnosed cases of TB worldwide [1,2]. Human immunodeficiency virus (HIV) and TB comorbidity, malnutrition, age, and gender can affect TB treatment outcomes. Males were more likely than females to have TB, with the incidence of the disease in elderly people being two to three times higher than in young people [3].

The 2021 WHO report stated that the incidence of TB in Somalia rose from 258 cases per 100,000 people in 2018 to 259 cases per 100,000 people in 2020. However, the death rate in Somalia was 68 per 100,000 people. The treatment of TB is affected by the emergence of MDR-TB. A recent WHO research estimates that 490,000 cases of multidrug-resistant bacteria (MDR-TB) occur worldwide annually [4,5]. According to several studies, it was found that the risk factors for TB were related to smoking, living in substandard conditions, abusing alcohol, and HIV infection. Furthermore, long-term conditions such as diabetes mellitus are critical in the spread of tuberculosis [6].

To stop the disease from spreading, TB patients must receive early diagnosis and treatment. Every year, millions of people are diagnosed with TB and receive effective treatment, saving millions of deaths; yet, there are still significant gaps in diagnosis and care. In many TB-endemic countries, the prevalence of MDR-TB, which is referred to as strains of *Mycobacterium tuberculosis* that are resistant to at least two drugs, rifampicin and isoniazid, keeps rising. High TB mortality can be attributed to both MDR and extensively drug-resistant TB (XDR-TB). The incidence of TB has increased in sub-Saharan Africa in part due to HIV prevalence. Moreover, treatment outcomes for patients infected with more resistant strains of tuberculosis are much worse than those for patients with less drug-resistant strains [7-10]. The disease progresses more quickly from exposure to invasive tuberculosis in a malnourished area. Furthermore, there is a link between nutritional deficiency and a higher chance of dying or experiencing a return of the illness. The course of tuberculosis treatment is also impacted by malnutrition. The global battle against tuberculosis is hampered by poor feeding and eating practices, particularly in developing nations [11].

This study aimed to evaluate the TB treatment outcome and its determinants among patients who attended Hargeisa TB Hospital, Somaliland, from January 2019 to December 2023.

## Methods

**Study design and setting:** a retrospective review of the TB treatment register was conducted at Hargeisa TB Hospital, the sole facility for diagnosing and treating TB in Hargeisa, the capital and one of the most populous cities in Somaliland. This review spanned the period from January 2019 to December 2023 and aimed to evaluate TB treatment outcomes and identify associated factors among patients receiving treatment.

**Study population and sample size:** in this study, all TB patients who attended and registered at Hargeisa TB Hospital for treatment during the period of the study were included, and a total of 6,127 patients were registered, but only 6,069 TB patients participated.

**Data collection procedure:** the records of all registered TB patients at Hargeisa TB Hospital were included in this study. For this retrospective study, data were accessed between 29^th^ December 2024 and 15^th^ January 2025.

### Inclusion and exclusion criteria

**Inclusion criteria:** patients with a confirmed diagnosis of tuberculosis and complete clinical and demographic information were included in the study.

**Exclusion criteria:** patients misdiagnosed with tuberculosis and those with incomplete clinical or demographic information were excluded from the study.

**Study variables:** the treatment outcome of TB (success and failure) was the dependent variable, whereas sex, age, year, and residence were the independent variables.

**Data processing and analysis:** R 4.2.2 software was used for data analysis. Descriptive statistics were employed to outline the characteristics of the participants, while logistic regression (both bivariate and multivariable) was utilized to assess the associations between independent and outcome variables. Variables with a p-value of less than 0.25 in the bivariate analysis were incorporated into the multivariable logistic regression model for further analysis. A p-value < 0.05 was considered significant.

**Operational definitions:** the operational terms were defined using the guidelines from the National TB Control Program, Ministry of Health Development, Somaliland; the WHO definitions and treatment framework for TB, as well as the WHO's treatment outcome definitions [12].

### TB patient category

**Treatment outcome variables:** a successful TB treatment outcome is defined as a patient who has successfully concluded their therapy and been cured of tuberculosis or who has completed the treatment regimen without difficulties.

Unsuccessful TB treatment outcome is defined as any circumstance in which TB therapy does not lead to a cure or completion as intended. And according to the WHO, regarding treatment outcome definitions for tuberculosis [13], the following definitions are used in our study. Cured: if, at the end of the treatment, the patients test negative in their bacteriological tests. Completed: if patients had completed treatment without bacteriological results at the end of the treatment. Failed: if the patients remain TB positive while on treatment, or a TB patient who, at the end of five months into therapy, has a positive sputum smear or culture. Defaulted: TB patients who did not start treatment or who stopped therapy for at least two months. Died: a patient with TB who passes away for any cause, either before or during treatment.

**Ethical consideration:** the national ethical committee, along with the unit of research development of the Ministry of Health Development of Somaliland, granted ethical clearance with the reference TIX: WHC/AG/2: 1648/2024. All data were maintained confidentially and utilized solely for their intended purpose. Informed patient consent was not required because the study involved only anonymized, retrospective data, and the information analyzed included only sex, age, and clinical variables necessary for this study.

## Results

**Sociodemographic characteristics of study participants:** a secondary data set with a total of 6127 TB patients was collected from Hargeisa TB Hospital. Fifty-eight patients have been removed from the analysis due to misdiagnosis and missing treatment outcomes. The sociodemographic information of the remaining patients (n = 6,069) is shown in Table 1. The majority of patients were male (62.6%) compared to female (37.4%). In terms of age, the largest proportion of patients was under 20 years old (37.2%), followed closely by those aged 20-40 years (35.7%). Patients aged 40-60 and over 60 years old accounted for 16.7% and 10.4%, respectively. Most of the patients resided in urban areas (88.3%), with only 11.7% living in rural areas. The data also shows that the number of patients varied by year, with the highest number of cases reported in 2019 (22.4%) and the lowest in 2020 (18.3%).

**Table 1:** sociodemographic information of tuberculosis patients (2019-2023)

Variable	Category	Frequency	Percent
**Sex**	Female	2272	37.4
	Male	3797	62.6
**Age**	< 20	2256	37.2
	20-40	2168	35.7
	40-60	1014	16.7
	> 60	631	10.4
**Residence**	Rural	712	11.7
	Urban	5357	88.3
**Year**	2019	1362	22.4
	2020	1111	18.3
	2021	1192	19.6
	2022	1249	20.6
	2023	1155	19.0

**Clinical characteristics of study participants:** Table 2 shows the clinical characteristics of TB patients attending Hargeisa TB Hospital from 2019 to 2023. The majority of patients were new cases (94.0%), with a smaller proportion being relapsed (0.6%) or transferred in (5.3%). Regarding the type of TB, 56.8% had pulmonary TB, while 43.2% had extra-pulmonary TB. The X-ray results for most patients (78.8%) were suggestive of TB, while 21.2% had non-suggestive X-rays. The GeneXpert test results revealed that 57% of cases were not determined, 9.6% were negative, and 33.4% were positive. Treatment outcomes showed a high success rate, with 81.1% of patients having a successful outcome, while 18.9% experienced an unsuccessful outcome.

**Table 2:** clinical characteristics of tuberculosis patients (2019-2023)

Category	Variable	Frequency	Percent
**Type of Patient**	New	5707	94.0
	Relapse	39	0.6
	Transfer in	323	5.3
**Type of TB**	Extra Pulmonary	2619	43.2
	Pulmonary	3450	56.8
**X-Ray**	Not Suggestive	1286	21.2
	Suggestive X-Ray	4782	78.8
**GeneXpert Test**	Not Determined	3459	57.0
	Negative	584	9.6
	Positive	2026	33.4
**Treatment Outcome**	Successful	4924	81.1
	Unsuccessful	1145	18.9

**Figure 1:**
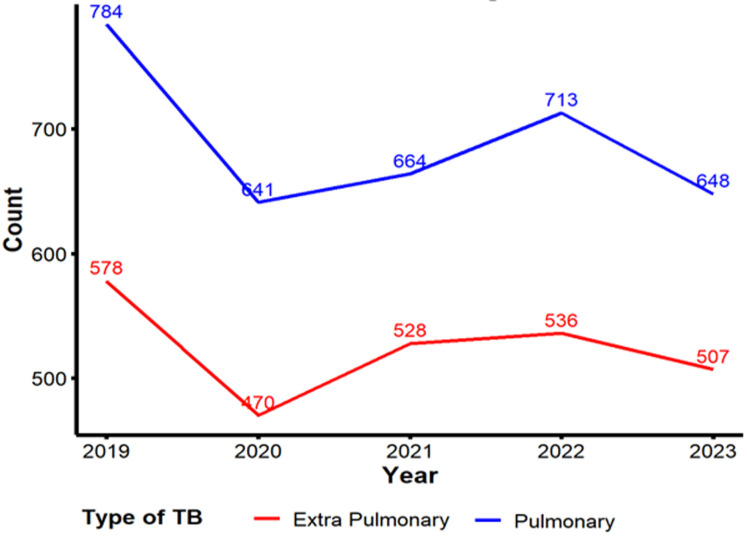
five-year trend of pulmonary and extra-pulmonary tuberculosis

**Five-year trend of pulmonary and extrapulmonary tuberculosis:** looking into the five-year trend (2019-2023) depicted in Figure 1, the number of pulmonary TB cases consistently surpassed those of extrapulmonary TB each year. pulmonary TB cases peaked in 2019 at 784, followed by a decline to 641 in 2020, before stabilizing around 664-713 in subsequent years. In contrast, extrapulmonary TB cases were highest in 2019 at 578 and followed a gradual decline, reaching 507 in 2023. The trends suggest a reduction in both extrapulmonary TB cases and pulmonary TB cases over time, though pulmonary TB consistently remains more prevalent, highlighting the need to sustain efforts in managing and preventing TB across both types.

**Figure 2:**
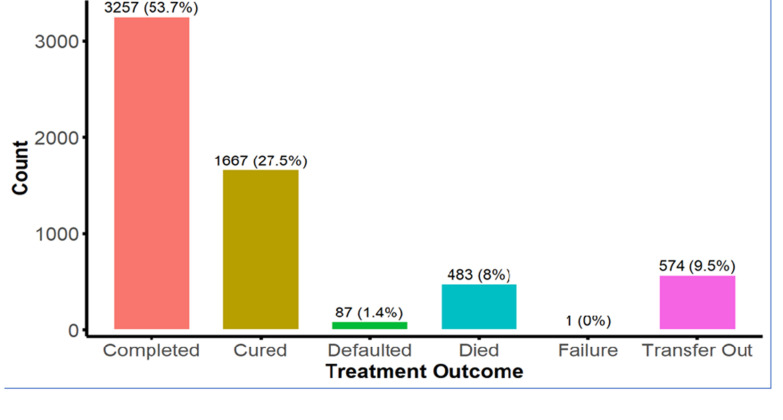
distribution of treatment outcome among tuberculosis patients

**Distribution of treatment outcome among TB patients:** Figure 2 illustrates the distribution of treatment outcome categories among TB patients, expressed in counts and percentages. The majority of patients (3257, approximately 54%) completed their treatment, followed by 1667 patients (27.5%) who were cured. However, a notable proportion transferred out to other hospitals, 574 patients (9.5%), while 483 patients (8%) succumbed to the disease, and 87 (1.4%) defaulted from treatment. Treatment failure was the least common outcome, affecting only 59 patients (1%). These results highlight that while treatment success rates (completion and cure) are relatively high, a significant proportion of patients experienced adverse outcomes, underscoring the need for interventions to reduce defaulting and mortality rates.

**Distribution of treatment outcome with sociodemographic and clinical characteristics of study participants:** we further investigated the distribution of detailed treatment outcome categories with sociodemographic and clinical characteristics of TB patients, and the results are presented in Table 3. The findings show that among all TB patients, 31.9% (n = 1,933) of the full sample were males who completed treatment, and 21.8% (n = 1,324) were females who completed treatment. Urban residents had a higher rate of treatment completion (47.3%) compared to rural residents (6.4%). New TB patients had the highest treatment completion rate (50.5%), while relapse and transfer-in patients had notably lower completion rates (0.3% and 2.9%, respectively). Pulmonary TB patients had a higher rate of cure (27.2%) compared to extrapulmonary TB patients (0.3%). HIV-negative patients had a significantly higher completion and cure rate (53.0%) compared to HIV-positive patients (0.7%). The data also show a gradual decrease in mortality and a gradual increase in default rates over the years, with 2019 having the highest number of deaths and the lowest number of defaults.

**Table 3:** distribution of treatment outcome with sociodemographic and clinical characteristics of tuberculosis patients

Variable	Category	Completed n (%)	Cured n (%)	Died n (%)	Defaulted n (%)	Failure n (%)	Transfer Out n (%)
**Sex**	Male	1933 (31.9)	1182 (19.5)	279 (4.6)	54 (0.9)	1 (0.0)	348 (5.7)
	Female	1324 (21.8)	485 (8.0)	204 (3.4)	33 (0.5)	0 (0.0)	226 (3.7)
**Age Group**	<20	1468 (24.2)	458 (7.5)	124 (2.0)	21 (0.3)	0 (0.0)	185 (3)
	20-40	1004 (16.5)	798 (13.1)	101 (1.7)	38 (0.6)	1 (0.0)	226 (3.7)
	40-60	479 (7.9)	291 (4.8)	131 (2.2)	16 (0.3)	0 (0.0)	97 (1.6)
	>60	306 (5.0)	120 (2.0)	127 (2.1)	12 (0.2)	0 (0.0)	66 (1.1)
**Residence**	Rural	389 (6.4)	177 (2.9)	66 (1.1)	6 (0.1)	0 (0.0)	74 (1.2)
	Urban	2868 (47.3)	1490 (24.6)	417 (6.9)	81 (1.3)	1 (0.0)	500 (8.2)
**Type of Patient**	New	3062 (50.5)	1564 (25.8)	458 (7.5)	83 (1.4)	1 (0.0)	539 (8.9)
	Relapse	21 (0.3)	11 (0.2)	4 (0.1)	0 (0.0)	0 (0.0)	3 (0.0)
	Transfer In	174 (2.9)	92 (1.5)	21 (0.3)	4 (0.1)	0 (0.0)	32 (0.5)
**Type of TB**	Pulmonary	1223 (20.2)	1651 (27.2)	240 (4.0)	51 (0.8)	0 (0.0)	285 (4.7)
	Extra Pulmonary	2034 (33.5)	16 (0.3)	243 (4.0)	36 (0.6)	1 (0.0)	289 (4.8)
**HIV Status**	Positive	42 (0.7)	28 (0.5)	26 (0.4)	3 (0.0)	0 (0.0)	13 (0.2)
	Negative	3215 (53.0)	1639 (27.0)	457 (7.5)	84 (1.4)	1 (0.0)	561 (9.2)
**Year**	2019	743 (12.2)	264 (4.3)	117 (1.9)	7 (0.1)	1 (0.0)	230 (3.8)
	2020	530 (8.7)	306 (5.0)	70 (1.2)	4 (0.1)	0 (0.0)	201 (3.3)
	2021	708 (11.7)	318 (5.2)	102 (1.7)	14 (0.2)	0 (0.0)	50 (0.8)
	2022	656 (10.8)	391 (6.4)	110 (1.8)	38 (0.6)	0 (0.0)	54 (0.9)
	2023	620 (10.2)	388 (6.4)	84 (1.4)	24 (0.4)	0 (0.0)	39 (0.6)

**Bivariate analysis of treatment outcome with sociodemographic and clinical characteristics of TB patients:** to assess the association between treatment outcome and different sociodemographic and clinical characteristics, we conducted a bivariate logistic regression analysis. The results are shown in Table 4. The analysis reveals several significant associations with treatment success. Female patients had lower odds of successful treatment compared to males (COR: 0.86, 95% CI: 0.75-0.98, p = 0.019). Younger age groups (<20 and 20-40) had significantly higher odds of successful treatment, with the odds decreasing as age increased, with the >60 age group serving as the reference (COR: 2.81, 95% CI: 2.29-3.44, p<0.001 for <20). Urban residence showed no significant effect on treatment outcome (p = 0.234). New TB patients had higher odds of success compared to relapse or transfer-in patients, with relapse and transfer-in having odds ratios close to 1, indicating no significant association with success. Pulmonary TB patients had significantly higher odds of treatment success compared to extrapulmonary TB patients (COR: 0.72, 95% CI: 0.63-0.82, p<0.001). HIV-negative patients had much higher odds of successful treatment compared to HIV-positive patients (COR: 2.64, 95% CI: 1.78-3.87, p<0.001). Finally, treatment success significantly improved over the years, with the highest odds ratios in 2021, 2022, and 2023, indicating a positive trend in treatment outcomes over time.

**Table 4:** bivariate analysis of treatment outcome with sociodemographic and clinical characteristics of tuberculosis patients

Variable	Category	Treatment Outcome		COR (95% CI)	P-value
-	-	Successful n (%)	Unsuccessful n (%)	-	-
**Sex**	Male	3115 (50.8)	682 (11.2)	1	-
	Female	1809 (29.5)	463 (7.6)	0.86 (0.75-0.98)	0.020
**Age**	<20	1926 (31.4)	330 (5.4)	2.81 (2.29-3.44)	0.000
	20-40	1802 (29.4)	366 (6.0)	2.37 (1.94-2.89)	0.000
	40-60	770 (12.6)	244 (4.0)	1.52 (1.22-1.89)	0.000
	>60	426 (7.0)	205 (3.4)	1	-
**Residence**	Rural	566 (9.2)	146 (2.4)	1	-
	Urban	4358 (71.1)	999 (16.5)	1.13 (0.92-1.36)	0.234
**Type of Patient**	New	4626 (75.5)	1081 (17.8)	1	-
	Relapse	32 (0.5)	7 (0.1)	1.07 (0.50-2.64)	0.875
	Transfer In	266 (4.3)	57 (0.9)	1.09 (0.82-1.48)	0.563
**Type of TB**	Pulmonary	2874 (46.9)	576 (9.5)	1	-
	Extra Pulmonary	2050 (33.5)	569 (9.4)	0.72 (0.63-0.82)	0.000
**HIV Status**	Positive	70 (1.1)	42 (0.7)	1	-
	Negative	4854 (79.2)	1103 (18.2)	2.64 (1.78-3.87)	0.000
**Year**	2019	1007 (16.4)	355 (5.8)	1	-
	2020	836 (13.6)	275 (4.5)	1.07 (0.89-1.29)	0.456
	2021	1026 (16.7)	166 (2.7)	2.18 (1.78-2.68)	0.000
	2022	1047 (17.1)	202 (3.3)	1.83 (1.51-2.22)	0.000
	2023	1008 (16.5)	147 (2.4)	2.42 (1.96-2.99)	0.000

**Multivariate analysis of treatment outcome with sociodemographic and clinical characteristics of TB patients:** to confirm the results of the bivariate analysis and adjust for confounding variables, we assessed the associations using multivariate logistic regression analysis. Table 5 shows the results of this analysis. The findings indicate that age is a significant factor in treatment success, with younger patients (<20 years) showing a higher likelihood of success (AOR= 2.89, 95% CI: 2.35-3.56), followed by patients aged 20-40 (AOR= 2.43, 95% CI: 1.98-2.98) and 40-60 (AOR= 1.54, 95% CI: 1.23-1.93). The odds of treatment success are lower for females (AOR= 0.88, 95% CI: 0.76-1.00), although this result is marginally non-significant (p = 0.056). Urban residence does not significantly affect treatment outcome (AOR= 1.10, 95% CI: 0.89-1.34, p = 0.505). Patients with extrapulmonary TB have lower odds of successful treatment compared to those with pulmonary TB (AOR= 0.75, 95% CI: 0.66-0.86). HIV-negative patients are more likely to have a successful outcome (AOR= 2.75, 95% CI: 1.82-4.10), and the likelihood of success has increased progressively over the years, with the year 2023 showing the highest odds (AOR= 2.66, 95% CI: 2.14-3.30). Relapse patients have slightly higher odds of success (AOR= 2.16, 95% CI: 0.97-5.47), but this is not statistically significant (p = 0.067), while transfer-in patients do not differ significantly from new patients (AOR= 1.02, 95% CI: 0.76-1.41).

**Table 5:** multivariate analysis of treatment outcome with sociodemographic and clinical characteristics of tuberculosis patients

Variable	Category	Treatment Outcome		AOR (95% CI)	P-value
	-	Successful n (%)	Unsuccessful n (%)	-	-
**Sex**	Male	3115 (50.8)	682 (11.2)	1	-
	Female	1809 (29.5)	463 (7.6)	0.88 (0.76-1.00)	0.056
**Age**	<20	1926 (31.4)	330 (5.4)	2.89 (2.35-3.56)	0.000
	20-40	1802 (29.4)	366 (6.0)	2.43 (1.98-2.98)	0.000
	40-60	770 (12.6)	244 (4.0)	1.54 (1.23-1.93)	0.000
	>60	426 (7.0)	205 (3.4)	1	-
**Residence**	Rural	566 (9.2)	146 (2.4)	1	-
	Urban	4358 (71.1)	999 (16.5)	1.10 (0.89-1.34)	0.372
**Type of Patient**	New	4626 (75.5)	1081 (17.8)	1	-
	Relapse	32 (0.5)	7 (0.1)	2.16 (0.97-5.47)	0.077
	Transfer In	266 (4.3)	57 (0.9)	1.02 (0.76-1.41)	0.897
**Type of TB**	Pulmonary	2874 (46.9)	576 (9.5)	1	-
	Extra Pulmonary	2050 (33.5)	569 (9.4)	0.75 (0.66-0.86)	0.000
**HIV Status**	Positive	70 (1.1)	42 (0.7)	1	-
	Negative	4854 (79.2)	1103 (18.2)	2.75 (1.82-4.10)	0.000
**Year**	2019	1007 (16.4)	355 (5.8)	1	-
	2020	836 (13.6)	275 (4.5)	1.10 (0.91-1.32)	0.329
	2021	1026 (16.7)	166 (2.7)	2.32 (1.89-2.87)	0.000
	2022	1047 (17.1)	202 (3.3)	1.93 (1.58-2.32)	0.000
	2023	1008 (16.5)	147 (2.4)	2.66 (2.14-3.30)	0.000

## Discussion

Evaluating TB treatment outcome and factors associated with unsuccessful treatment has a public health importance, and in this study, we aimed to assess treatment outcomes and determinants associated with unsuccessful treatment among attendants in Hargeisa TB Hospital, Hargeisa, Somaliland. In our study, the majority of TB patients were males (62.6%), and this is consistent with similar previous studies conducted in Ethiopia (60.2%) [13], in Somalia (64.58%) [14], in Kenya (60%) [15], in Uganda (68.9%) [16] and in Nigeria (59%) [17]. The fact that men are more likely than women to be exposed to the disease could be the cause of the greater TB rate among men.

The largest proportion of patients was under 20 years old (37.2%), followed by those aged 20-40 years (35.7%), while patients aged 40-60 and over 60 years old accounted for 16.7% and 10.4%, respectively. Most of the TB cases in our study were under 40 years of age, and this is similar to previous findings [1]. In our study, the majority of TB patients were in the new cases category, accounting for 94%, whereas 0.6% were in the relapse category, but 5.3% were transferred into Hargeisa TB Hospitals. In terms of new cases, similar findings were reported in Bossaso, Somalia 87% [18], and Harar, Ethiopia, 97% [1], and Nigeria, 88.8% [19]. The patients that presented extra-pulmonary TB (EPTB) in our study were 43.2%, and similar findings were reported in Addis Ababa, Ethiopia, 40.1% [20], and in Bossaso, Somalia, 36.1% [18]. This high proportion of EPTB in our study could be due to overdiagnosis or might be due to sample size differences. In our study, the overall successful TB treatment outcome was 81.2%, this finding was lower than successful TB treatment outcomes in Bossaso 88.5% [18], in Galkayo 85% [21], in southern Ethiopia 92.4% [22], and in Oromia region of Ethiopia 94.2% [23], in Mogadishu, Somalia 81.8% [24]. However, our finding was higher than successful TB treatment outcomes in Lesotho, 73.4% [25], in north eastern Uganda, 71.9% [26]. These differences might be due to variation in the socioeconomic status of respondents, access to TB healthcare centers, sample size, and study duration.

Identification of risk factors associated with TB treatment outcomes is very important in the public health aspect in order to intervene in the factors linked to poor treatment outcomes. The study revealed several significant associations with treatment success, and female, age, type of TB, and HIV status were linked with successful treatment outcomes. In our study, we found that age groups were significantly associated with treatment outcomes. Younger TB patients (<20 years) had significantly higher success rates, followed by patients between 20 and 40 years. As the age of the patients increases, the rate of successful treatment outcome decreases, in agreement with similar findings in Somalia, Kenya, and South Africa [18,15,27].

The study showed that HIV-negative patients had higher successful TB treatment outcomes compared to TB patients co-infected with HIV, similar to previous studies [28,29]. The reason why TB-HIV co-infected patients had poor TB treatment outcomes was due to the decreased immune status of HIV-positive patients and TB/HIV drug interactions, especially when drug-drug and drug-disease interactions were not carefully considered, resulting in unsuccessful TB treatment outcomes [30,31]. The current study showed that pulmonary tuberculosis (PTB) patients had significantly higher odds of treatment success compared to EPTB patients, and this is in line with previous studies in Ethiopia [22,32]. The reasons why EPTB has poor treatment outcomes when compared to those of pulmonary tuberculosis could be due to the lower positive rate in diagnosis and less public health awareness required [33].

**Limitation:** this study has several limitations inherent to retrospective secondary data analysis. Missing or incomplete information related to clinical, diagnostic, and demographic variables may have affected the accuracy of treatment outcome assessment. Additionally, important confounding factors such as socioeconomic status and nutritional status were not available in the dataset and could not be accounted for in the analysis. Despite these limitations, this study offers valuable insights into TB treatment outcomes in the context of the study setting.

## Conclusion

In this study, the overall TB treatment outcome was 81.2%, and advanced age and HIV-positive patients were associated with lower TB treatment outcomes. The study suggests that public health awareness, early detection and treatment of cases should be improved, and further research is also required to find out factors in association with poor treatment outcomes. The study suggests strengthening diagnostic accuracy to prevent misdiagnosis, particularly among cases where treatment is terminated, as such errors can undermine TB management and control.

### What is known about this topic

- Globally, tuberculosis continues to be a major infectious cause of morbidity and mortality, particularly in developing countries;
- Key performance indicators for TB control programs are treatment outcomes, and TB-HIV co-infection and old age are associated factors with unfavorable TB treatment outcomes.

### What this study adds

- The overall tuberculosis treatment success rate at Hargeisa TB Hospital was below the WHO target;
- The study reveals a significantly high mortality rate among TB patients, indicating critical gaps in treatment adherence and follow-up systems;
- Patients under 20 years of age demonstrated higher treatment success rates.
